# Altered local connectivity in chronic pain: A voxel-wise meta-analysis of resting-state functional magnetic resonance imaging studies

**DOI:** 10.1097/MD.0000000000021378

**Published:** 2020-07-31

**Authors:** XiaoGuang Lin, Dan Zhen, HuaLiang Li, JianGuo Zhong, ZhenYu Dai, CongHu Yuan, PingLei Pan

**Affiliations:** a Department of Neurology, The Affiliated Suqian Hospital of Xuzhou Medical University, Suqian, Jiangsu; b Jiangsu Vocational College of Medicine; c Department of Neurology; d Department of Radiology; e Department of Anesthesia and Pain Management; f Department of Central Laboratory, Affiliated Yancheng Hospital, School of Medicine, Southeast University, Yancheng, P.R. China.

**Keywords:** chronic pain, coordinate-based meta-analysis, regional homogeneity, resting-state functional MRI, Seed-based *d* Mapping

## Abstract

**Background:**

A number of studies have used regional homogeneity (ReHo) to depict local functional connectivity in chronic pain (CP). However, the findings from these studies were mixed and inconsistent.

**Methods:**

A computerized literature search will be performed in PubMed, Web of Science, Embase, China National Knowledge Infrastructure (CNKI), WanFang, and SinoMed databases until June 15, 2019 and updated on March 20, 2020. This protocol will follow the Preferred Reporting Items for Systematic review and Meta-Analysis Protocols (PRISMA-P). The Seed-based *d* Mapping with Permutation of Subject Images (SDM-PSI) software will be used for this voxel-wise meta-analysis.

**Results:**

This meta-analysis will identify the most consistent ReHo alterations in CP.

**Conclusions:**

To our knowledge, this will be the first voxel-wise meta-analysis that integrates ReHo findings in CP. This meta-analysis will offer the quantitative evidence of ReHo alterations that characterize brain local functional connectivity of CP.

**PROSPERO registration number:**

CRD42019148523

## 1 Introduction

Chronic pain (CP) is a public health issue worldwide with an estimated prevalence of 31% in the general population.^[1,2]^ CP is now recognized as a complex multifactorial disease entity. Biological and psychosocial risk factors, comorbidities, and pain-processing mechanisms may contribute to the pathogenesis of CP.^[3–5]^ Converging evidence suggests that central sensitization is a common pathophysiological process that has been implicated in CP conditions,^[6]^ which is associated with brain structural and functional reorganization.^[7–13]^

Resting-state functional magnetic resonance imaging (RS-fMRI) measures the brain blood oxygenation level-dependent (BOLD) signal during rest, reflecting the intrinsic functional organization of the brain.^[14]^ RS-fMRI enables researchers not only to investigate long-distance interregional functional connectivity (FC), but also to examine local short-range FC in the human brain, which contribute to modeling the human brain connectome.^[14,15]^ Regional homogeneity (ReHo) is a popular approach to depict local FC, which estimates the similarity of the BOLD signal in a single voxel with its nearest neighboring voxels.^[16]^ ReHo has been widely used in many neuropsychological disorders.^[17–24]^

In recent years, many studies have used ReHo to investigate local FC in CP and bring insights into its pathophysiology. Although expanding our current understanding regarding the neural basis of CP,^[25–31]^ the findings from these studies were mixed and inconsistent. Meta-analysis is therefore required to increase the statistical power to quantitatively elucidate whether there is a consistent and common pattern of local FC alterations in CP. In this study, we performed a coordinate-based meta-analysis (CBMA) using Seed-based *d* Mapping with Permutation of Subject Images (SDM-PSI)^[32]^ to identify the most consistent ReHo abnormalities in CP.

## 2 Methods

### 2.1 Search strategies

We will search the following electronic databases: PubMed, Embase, and Web of Science from each database's inception to June 15, 2019 and updated on March 20, 2020 for records published in English. We will also search China National Knowledge Infrastructure (CNKI), WanFang, and SinoMed databases for records published in Chinese. We will use both general and specific CP keywords: (“pain” OR “chronic pain” OR “chronic widespread pain” OR “chronic musculoskeletal pain” OR “chronic headache” OR “chronic migraine” OR “chronic tension-type headache” OR “trigeminal neuralgia” OR “chronic myofascial pain” OR “chronic burning mouth syndrome” OR “neuropathic pain” OR “temporomandibular disorder∗” OR “chronic neck pain” OR “chronic shoulder pain” OR “chronic thoracic pain” OR “chronic chest pain” OR “chronic back pain” OR “chronic knee pain” OR “chronic ankle pain” OR “chronic limb pain” OR “chronic abdominal pain” OR “chronic visceral pain” OR “chronic pelvic pain syndrome” OR “complex regional pain syndrome” OR “fibromyalgia” OR “chronic fatigue syndrome” OR “osteoarthritis” OR “arthritis” OR “chronic whiplash-associated disorder” OR “chronic epicondylalgia∗” OR “postherpetic neuralgia” OR “ankylosing spondylitis” OR “chronic epigastric pain syndrome” OR “irritable bowel syndrome” OR “inflammatory bowel disease” OR “Crohn disease” OR “dysmenorrhea” OR “chronic bladder pain syndrome” OR “chronic testicular pain” OR “functional dyspepsia” OR “somatoform pain”) AND (“regional homogeneity” OR “ReHo” OR “local connectivity”).

We will also manually examine the reference lists of the included studies and relevant review articles and meta-analyses for additional related studies for potential inclusion. This protocol will follow the Preferred Reporting Items for Systematic review and Meta-Analysis Protocols (PRISMA-P).^[33]^

### 2.2 Eligibility criteria

A study will be included if it conforms all of the following criteria: the study should report original data published in a peer-reviewed scientific journal; individuals included in the study should be human patients with CP, which was defined as persistent or recurrent pain that lasted longer than 3 months^[34]^; the study should use a whole-brain voxel-wise ReHo analysis to investigate local connectivity differences between patients with CP and pain-free control subjects; and the study should report the imaging results in standard Montreal Neurological Institute (MNI) or Talairach space.

**Figure 1:**
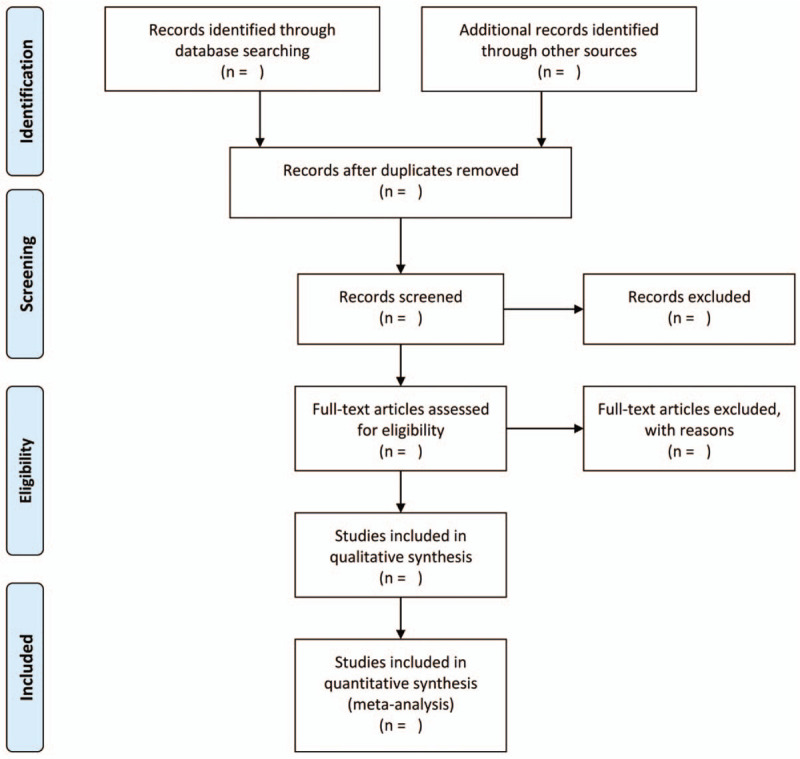
Study selection process following the PRISMA flowchart. PRISMA = Preferred Reporting Items for Systematic review and Meta-Analysis.

We will exclude publications if individuals included in the study did not meet the definition of CP, like those with episodic headaches,^[35]^ acute pain, and experimental pain; sample sizes in each group were <7 subjects; no peak stereotactic coordinates were available; they lacked a direct group comparison between patient with CP and pain-free controls; longitudinal/interventional studies did not report baseline comparison results; the studies only applied regions of interest or surface-based imaging or small volume correction analyses; and they were conference abstracts, editorial letters, case reports, reviews, or meta-analyses. We will select the study with a larger sample size in case of studies that used overlapping samples. The study selection process flowchart will be presented in Fig. 1.

### 2.3 Data extraction

We will extract the following information from each included study: The name of the first author, publication year, sample size, percentage of women, mean age, patient type, pain intensity (indexed using the visual analog scale [VAS]), pain duration, field strength of MRI scanner, smooth kernel, and statistical threshold for the ReHo analysis, peak coordinates, their corresponding effect sizes (*t* statistic, *z* score, *P*-value, etcetera), and their stereotactic reference space (Talairach or MNI).

### 2.4 Quality assessment

A 20-point checklist based on previous rs-fMRI meta-analyses^[21]^ will be used for quality assessment of the studies included (details in Table 1).

**Table 1:**
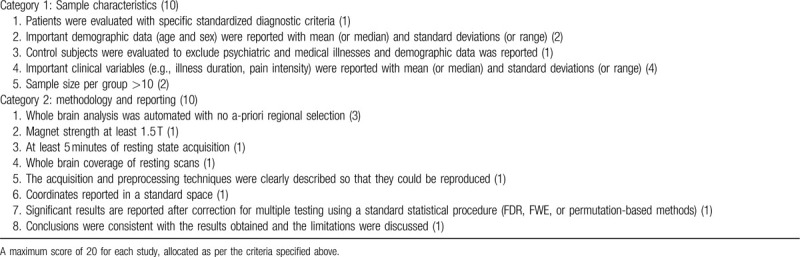
Criteria for objective assessment of methodological quality of individual studies.

Two independent authors (XGL and DZ) will perform the literature search, study selection, data extraction, and quality assessment. Any discrepancies will be resolved by discussion with consensus.

### 2.5 Main coordinate-based meta-analysis

The SDM-PSI software package (version 6.21, https://www.sdmproject.com/) will be used for this CBMA to identify consistent local connectivity differences between patients with CP and pain-free control subjects. The steps of SDM-PSI have been described in detail elsewhere.^[32,36]^ A recommended statistical threshold of threshold-free cluster enhancement-based family-wise error (FWE) correction for multiple comparisons *P* < .05, and voxel extent ≥10 will be applied.^[32,36]^

### 2.6 Reliability analysis

To test the stability of the results, we will perform a jackknife sensitivity analysis by iteratively repeating the same analysis *K* – 1 times (*K* = the number of datasets included), discarding one dataset each time. Clusters that are present in at least 80% of the jackknife folds indicate robustness.

We will extract the information of the peak MNI coordinate reported in the main CBMA to derive standard heterogeneity statistics *I*^2^. An *I*^2^ < 50% indicates low heterogeneity.

Egger tests for detection of the potential publication bias will be used. An Egger test with *P* < .05 is considered significant.

### 2.7 Subgroup analysis

Subgroup CBMA will be performed in clinical subtypes and imaging methodology variables if the corresponding number of the datasets is sufficient (n ≥ 10).

### 2.8 Meta-regression analysis

Exploratory meta-regression analyses will be performed to estimate which demographic and clinical variables could potentially lead to heterogeneity across studies. All results will be thresholded using the threshold-free cluster enhancement (TFCE)-based FWE corrected threshold (*P* < .05, voxel extent ≥10).^[32,36]^

### 2.9 Ethics and dissemination

Ethics approval is not required because this meta-analysis will be performed using the data based on published studies. The results of this meta-analysis will be published in a peer-reviewed scientific journal.

## 3 Discussion

Converging evidence suggests that central sensitization is a common pathophysiological process that has been implicated in CP conditions,^[6]^ which is associated with brain structural and functional reorganization.^[7–13]^ RS-fMRI has been widely applied in neuropsychiatric disorders and expanded our understanding of the human brain in the past 2 decades. However, replicability crisis is always a challenge in neuroimaging research.^[37]^ This crisis may be attributed to underpowered small sample sizes, phenotypic heterogeneity in CP conditions, and/or the use of different imaging protocols. Using a CBMA approach, we aimed to identify consistent ReHo alterations in CP. The findings will help to better understand of the commonalities of central mechanisms of this disease entity.

## Author contributions

**Conceptualization:** XiaoGuang Lin, CongHu Yuan, PingLei Pan

**Data curation:** XiaoGuang Lin, Dan Zhen

**Formal analysis:** XiaoGuang Lin, HuaLiang Li

**Funding acquisition:** PingLei Pan

**Investigation:** XiaoGuang Lin, Dan Zhen, JianGuo Zhong, HuaLiang Li

**Methodology:** ZhenYu Dai, JianGuo Zhong, PingLei Pan

**Project administration:** CongHu Yuan, PingLei Pan

**Resources:** XiaoGuang Lin, Dan Zhen, JianGuo Zhong, HuaLiang Li

**Software:** ZhenYu Dai, JianGuo Zhong, PingLei Pan

**Supervision:** CongHu Yuan, PingLei Pan

**Validation:** PingLei Pan

**Visualization:** XiaoGuang Lin, Dan Zhen, HuaLiang Li

**Writing – original draft:** XiaoGuang Lin, Dan Zhen

**Writing – review & editing:** ZhenYu Dai, CongHu Yuan, PingLei Pan
